# Candidate gene prioritization for chronic obstructive pulmonary disease using expression information in protein–protein interaction networks

**DOI:** 10.1186/s12890-021-01646-9

**Published:** 2021-09-04

**Authors:** Wan Li, Yihua Zhang, Yahui Wang, Zherou Rong, Chenyu Liu, Hui Miao, Hongwei Chen, Yuehan He, Weiming He, Lina Chen

**Affiliations:** 1 College of Bioinformatics Science and Technology, Harbin Medical University, Harbin, 150000 Heilongjiang China; 2 Institute of Opto-Electronics, Harbin Institute of Technology, Harbin, 150000 Heilongjiang China

**Keywords:** Chronic obstructive pulmonary disease, Candidate gene prioritization, Expression information, Protein–protein interaction networks

## Abstract

**Background:**

Identifying or prioritizing genes for chronic obstructive pulmonary disease (COPD), one type of complex disease, is particularly important for its prevention and treatment.

**Methods:**

In this paper, a novel method was proposed to Prioritize genes using Expression information in Protein–protein interaction networks with disease risks transferred between genes (abbreviated as PEP). A weighted COPD PPI network was constructed using expression information and then COPD candidate genes were prioritized based on their corresponding disease risk scores in descending order.

**Results:**

Further analysis demonstrated that the PEP method was robust in prioritizing disease candidate genes, and superior to other existing prioritization methods exploiting either topological or functional information. Top-ranked COPD candidate genes and their significantly enriched functions were verified to be related to COPD. The top 200 candidate genes might be potential disease genes in the diagnosis and treatment of COPD.

**Conclusions:**

The proposed method could provide new insights to the research of prioritizing candidate genes of COPD or other complex diseases with expression information from sequencing or microarray data.

**Supplementary Information:**

The online version contains supplementary material available at 10.1186/s12890-021-01646-9.

## Background

Chronic obstructive pulmonary disease (COPD) is a public health problem causing morbidity and mortality [1]. As a multifactorial and polygenic disease, COPD is caused by many factors, including smoking, advanced age, systemic inflammation, and especially pathways or processes influenced by protein–protein interactions (PPIs), such as oxidative stress and protease activity affected by interactions between glutathione S-transferase M1 and matrix metalloproteinases 1, 9, and 12 in the pathogenesis of COPD [2]. Identification or prioritizing COPD candidate genes is particularly important for its prevention and treatment.

Computational methods for disease candidate gene prioritizing has been conducted in terms of PPI networks. The accuracy/performance of these methods was evaluated by the rank of known disease genes in their ranked lists, reflecting their ability to recognize known disease genes from other genes. If many known disease genes are ranked highly, the Area Under the Curve (AUC) value is large, which calculated from plotting the Receiver Operation Characteristic (ROC) curves using Leave One Out Cross-Validation (LOOCV), a widely used method in many existing works [3, 4]. Methods with AUC > 0.70 were accurate or with good performance. For example, the online tool ToppNet of the ToppGene Suite (https://ToppGene.cchmc.org) [5] and another method proposed by Razaghi-Moghadam et al. [6] prioritized candidate genes employing the topological measures of PPI networks. Ganegoda et al. prioritized candidate genes of several types of cancer by evaluating similarity between diseases and similarity between proteins in a PPI network [7]. The random walk method considering probability transition was often used [8, 9], and was employed in the Random Walk and k-step Markov algorithms of LynxKB (http://lynx.ci.uchicago.edu/), a database and knowledge extraction engine for integrative medicine [10]. A random walk-based computational method was developed to prioritize ectopic pregnancy-related genes based on text mining data and PPI network information [11].

Although these current approaches have achieved good performance, improvements through integrating other information are still necessary. Expression information could reveal differential expression pattern between normal and disease samples. In our previous work, functional information has been integrated to a COPD-related PPI network to prioritize candidate genes [12]. FUN-L (http://funl.org/) is a tool for prioritizing genes by their probability of sharing pathways to a set of query genes [13]. ToppGene, another tool of the ToppGene Suite, prioritized candidate genes based on similarities of comprehensive factors, such as network topology, functional annotation and expression information. Network propagation has been used in this type of tools to compute the influence of initial vertexes (or disease genes) to other vertexes for gene prioritization [14, 15]. Borrowing ideas from network propagation, the influence of genes to another was referred to as disease risks transferred between each other in this study. A novel method was proposed to Prioritize genes using Expression information in PPI networks with disease risks transferred between genes. Our method was named PEP as the acronym of three key words (the first letter capitalized) from the description of the method. In the PEP method, a weighted COPD PPI network was constructed using expression information and then disease candidate genes were prioritized based on their corresponding disease risk scores in descending order. To evaluate the performance of the PEP method, AUC values were calculated using LOOCV and compared for different parameters and for different methods, as other researches did. The PEP method could prioritize candidate genes of COPD effectively and robustly with expression information from sequencing or microarray data.

## Methods

### Data

COPD disease genes were derived from Online Mendelian Inheritance in Man (OMIM, http://omim.org/) [16], which contained 29 genes. PPIs for products of COPD disease genes were obtained from the STRING database Version 10.0 (http://string-db.org/) [17]. After filtering out duplicated PPIs and merging transcripts matching to the same gene to one vertex (labeled by the official gene symbol), a COPD PPI network comprising products of 3740 genes (vertexes) and 7792 interactions (edges) were constructed. Non-COPD disease genes were candidate genes to be prioritized.

Gene expression information was retrieved from the Gene Expression Omnibus (GEO) database at the National Centre for Biological Information (NCBI) through accession number GSE57148 (https://www.ncbi.nlm.nih.gov/geo/query/acc.cgi?acc=GSE57148), which was an RNA-seq profiling of lung tissue in 98 COPD patients and 91 healthy controls [18]. As introduced in the original paper, the inclusion criteria were a postbronchodilator FEV1/FVC ratio (ratio of forced expiratory volume in the first second to forced vital capacity) of less than 0.7 for the COPD group and normal spirometry for the control group in accordance with American Thoracic Society/European Respiratory Society criteria (Table 1, mean ± standard deviation is shown).

**Table 1:** Demographics of COPD and control subjects for GSE57148

	COPD subjects	Control subjects
Male, n (%)	98,100.0	91,100.0
Age, years	67.5 ± 6.4	60.9 ± 9.5
Smoking (py)	48.0 ± 22.0	35.2 ± 17.2
FEV1, %	71.9 ± 13.4	91.0 ± 12.4
FEV1/FVC	57.1 ± 7.8	74.8 ± 4.3
DLCO, %	77.4 ± 13.8	92.8 ± 13.2

py: pack-years; FEV1: forced expiratory volume in 1 s; FVC: forced vital capacity; DLCO: diffusing capacity of the lung for CO_2_

### The PEP method

The PEP method was conducted in two steps.

#### Construction of the weighted COPD PPI network

In the COPD PPI network, vertex weights and edge weights were calculated using gene expression information.

Vertex weight $w\left( v \right)$ for vertex $v$ was computed as the differential of expression values in different samples as follows:

$$w(v) = \frac{{\overline{{E_{{v_{D} }} }} }}{{\overline{{E_{{v_{N} }} }} }}$$

where $\overline{{E_{{v_{D} }} }}$ and $\overline{{E_{{v_{N} }} }}$ represent the average of expression values for gene $v$ in disease samples and normal samples, respectively.

Edge weight $w\left( {u,v} \right)$ was defined as the Pearson correlation coefficient between vertex $u$ and vertex $v$ in the state of COPD, as follows:

$$w\left( {u,v} \right) = \frac{{\overline{{E_{u} E_{v} }} - \overline{{E_{u} }} \overline{{E_{v} }} }}{{\sqrt {\overline{{E_{u}^{2} }} - \left( {\overline{{E_{u} }} } \right)^{2} } \sqrt {\overline{{E_{v}^{2} }} - \left( {\overline{{E_{v} }} } \right)^{2} } }}$$

where $E_{u}$ and $E_{v}$ represent expression values of COPD patients for gene $u$ and gene $v$, and $\overline{X}$ is the average of the values in $X$.

Integrating all the above weights, a weighted COPD PPI network was constructed.

#### Prioritization of candidate genes with gene disease risk scores

The gene disease risk scores $S$ were calculated to evaluate the disease risks of genes using the strategy of disease risks transferred between genes with an iteration process, which was performed until the difference between $S^{\left( i \right)}$ and $S^{{\left( {i + 1} \right)}}$ was less than $10^{ - 8}$.

$$S^{{\left( {i + 1} \right)}} = \left( {1 - k} \right)TS^{\left( i \right)} + kS^{\left( 0 \right)}$$

where $S^{{\left( {i + 1} \right)}}$ is the vector of gene disease risk scores of all genes in the COPD PPI network at step $i$, and $k \in \left[ {0,1} \right]$ is a value measuring the importance between vertexes and edges.

The initial disease risk score vector $S^{\left( 0 \right)}$ is composed of initial scores $s$ s for all vertexes. The score $s\left( v \right)$ for vertex $v$ was defined as

$$s\left( v \right) = \left\{ {\begin{array}{*{20}c} {\frac{h \cdot w\left( v \right)}{{\sum\nolimits_{a \in G} {h \cdot w\left( a \right)} + \sum\nolimits_{a \notin G} {w\left( a \right)} }},} & {\quad v \in G} \\ {\frac{w\left( v \right)}{{\sum\nolimits_{a \in G} {h \cdot w\left( a \right)} + \sum\nolimits_{a \notin G} {w\left( a \right)} }},} & {\quad v \notin G} \\ \end{array} } \right.$$

where $G$ represents the set of COPD disease genes, $w\left( v \right)$ represents the vertex weight of $v$, and $h$ is an integer parameter to measure the significance of the COPD disease genes and candidate genes.

The disease risk transition score matrix $T$ is made up of the transition scores $t$ s. The formula for the disease risk transition score $t\left( {v|u} \right)$ from vertex $u$ to vertex $v$ was as follows:

$$t\left( {v|u} \right) = \frac{{w\left( {u,v} \right)}}{{\sum\nolimits_{{r \in {\text{neighbor}}\left( u \right)}} {w\left( {u,r} \right)} }}$$

where $w\left( {u,v} \right)$ represents the weight of the edge between vertex $u$ and vertex $v$, and ${\text{neighbor}}\left( u \right)$ represents the set of vertexes interacting with vertex $u$. If vertex $u$ has no neighbors, $t\left( {v|u} \right)$ is 0.

The genes in the weighted COPD PPI network were prioritized based on their corresponding disease risk scores $S$ in descending order. Top ranked genes were more related to the disease.

#### Parameter optimal value determination

The performance of the PEP method was evaluated and compared by AUC values using LOOCV based on COPD disease gene from the OMIM database for different parameters, $h$ and $k$, to determine their optimal values. In each round of LOOCV, one COPD disease gene was selected as a test gene, while other COPD disease genes in the weighted COPD PPI network were used to prioritize the candidate genes and the test gene. This process was repeated until all COPD disease genes were set as test genes. Sensitivity (frequency of test genes that were ranked above a particular threshold) and specificity (the percentage of test genes ranked below the threshold) were calculated. ROC curves were plotted based on the sensitivity versus 1-specificity (true versus false positive rate) of the test genes by varying the threshold. The AUC value was then measured to facilitate the comparison for different parameters. The performance with AUC = 1 is perfect since all test genes are ranked first in their respective ranked list, with AUC = 0.5 is no better than a random prioritization, with AUC > 0.5 is better than the random one, and with AUC < 0.5 is a worse one. The AUC is large when many disease genes are ranked highly. Thus, optimal parameter values with the highest AUC value were determined for the PEP method. The PEP method was effective if the AUC > 0.70 with optimal parameter values.

### Evaluation of performance

In order to demonstrate the robustness of the PEP method, The AUC values of LOOCV were used to evaluate the performance for random sample sets. Four sets of samples were randomly selected from the original RNA-seq profiling—20, 60, 100 and 140 samples—with an equal number of disease samples and of normal samples (10, 30, 50 and 70). The randomization process was repeated 100 times. Then, for each sample set, AUC values of LOOCV were calculated.

LOOCV was also performed to compare the performance of the PEP method with that of other state-of-the-art network-based prioritization methods, including ToppNet of the ToppGene Suite, Random Walk and k-step Markov of LynxKB, which exploited the topological information, as well as our previous work in [12], ToppGene and FUN-L exploiting functional information.

To prove the relationships of the top 200 candidate genes and COPD, a literature review was performed. Then to further verify their relevance, functional enrichment analysis was conducted for the top 200 candidate genes employing the Database for Annotation, Visualization and Integrated Discovery (DAVID) v6.8 (https://david.ncifcrf.gov/) [19, 20]. The Biological Process of Gene Ontology (GO) functions and pathways of Kyoto Encyclopedia of Genes and Genomes (KEGG) with FDR < 0.05 were statistically significant.

Moreover, to further exhibit the effectiveness and robustness of the PEP method, it was applied to another independent microarray data, GSE76925 from the GEO database, which contained 40 normal samples and 111 COPD patients. The AUC values of LOOCV were calculated for all samples and different sample sizes (20, 40, 60 and 80 samples from GSE76925, in which the number of disease samples and normal samples was equal) as mentioned above, respectively.

## Results

### Parameters of the PEP method

#### Optimal value of parameter $h$

**Fig. 1:**
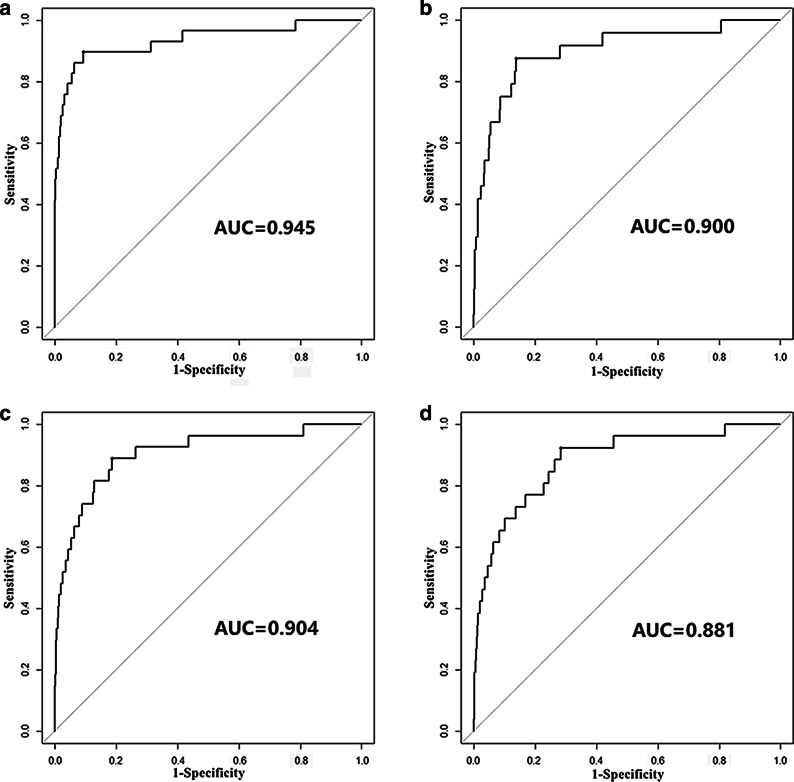
ROC curves for the PEP method with **a** $h = 1$, **b** $h = 10$, **c** $h = 15$ and **d** $h = 30$

Parameter $h$ was used to evaluate the significance of the COPD disease genes and candidate genes in the weighted COPD PPI network. LOOCV was used to investigate the performance for a range of $h$ values (1, 10, 15 and 30), which were shown as ROC curves (Fig. 1). The AUC values corresponding to these ROC curves (> 0.88) showed that good performance could be attained with all $h$ values. The best performance was achieved when $h = 1$, which implied that the COPD disease genes and candidate genes were of equal importance in the COPD PPI network.

#### Optimal value of parameter $k$

The $k$ parameter in the PEP method was used to evaluate the significance between vertexes and edges in the weighted COPD PPI network. LOOCV was also conducted to evaluate their performance for different $k$ values (Table 2).

**Table 2:** AUC values using different $k$ values of the PEP method

$k$	0	0.1	0.2	0.3	0.4	0.5	0.6	0.7	0.8	0.9	1
AUC	0.808	0.885	0.894	0.904	0.915	0.924	0.931	0.945	0.933	0.925	0.541

AUC values for $k$** = **0 or 1 were lower than those for other $k$ values. This demonstrated that the performance when the vertex and edge information were considered simultaneously was better than that when only the vertex or the edge information was considered. The AUC value was the highest for $k$** = **0.7, demonstrating that vertexes were more important than edges in the COPD PPI network.

As mentioned above, $h$ = 1 and $k$** = **0.7 were optimal parameter values for the PEP method with and AUC of 0.945, indicating the effectiveness of the PEP method. These optimal values were used to calculate the gene disease risk scores in the following sections.

### Assessment and comparison

#### Robustness assessment

**Fig. 2:**
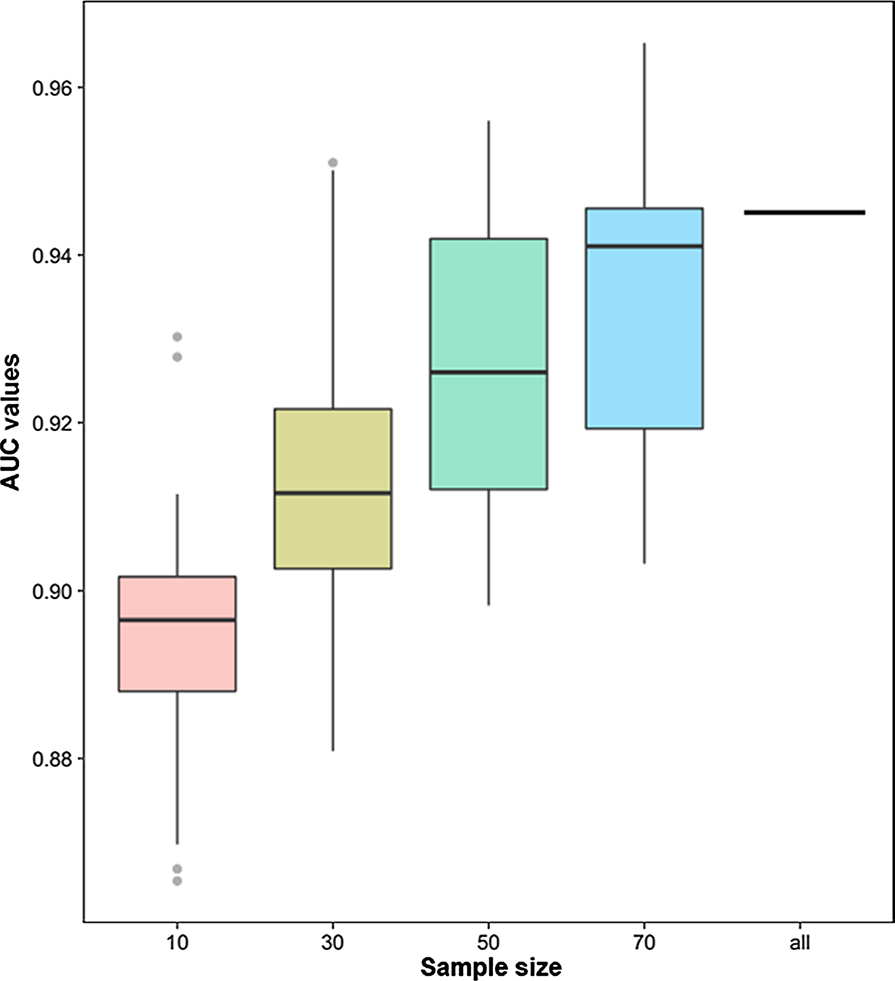
The distribution of AUC values for different sample sizes. Horizontal lines in each box plot from the bottom to the top are first quartile, median and third quartile, and dots are outliers

In the COPD PPI network, the vertex and edge weights were calculated using gene expression information from all samples in GSE57148. Here, in order to confirm that the results were not affected by various sample sizes, four sets of samples were randomly selected with an equal number of disease samples and normal samples. The randomization process was repeated 100 times. The AUC values of LOOCV were also used to evaluate their performance (Fig. 2). With the growth of the sample sizes, the AUC values also had a growth tendency. For different sample sizes, all AUC values were larger than 0.86, indicating the robustness of the PEP method. The AUC value for all samples was higher than any median of the AUC values for other sample sizes. Thus, using all samples was appropriate to prioritize candidate genes.

#### Method comparison

**Fig. 3:**
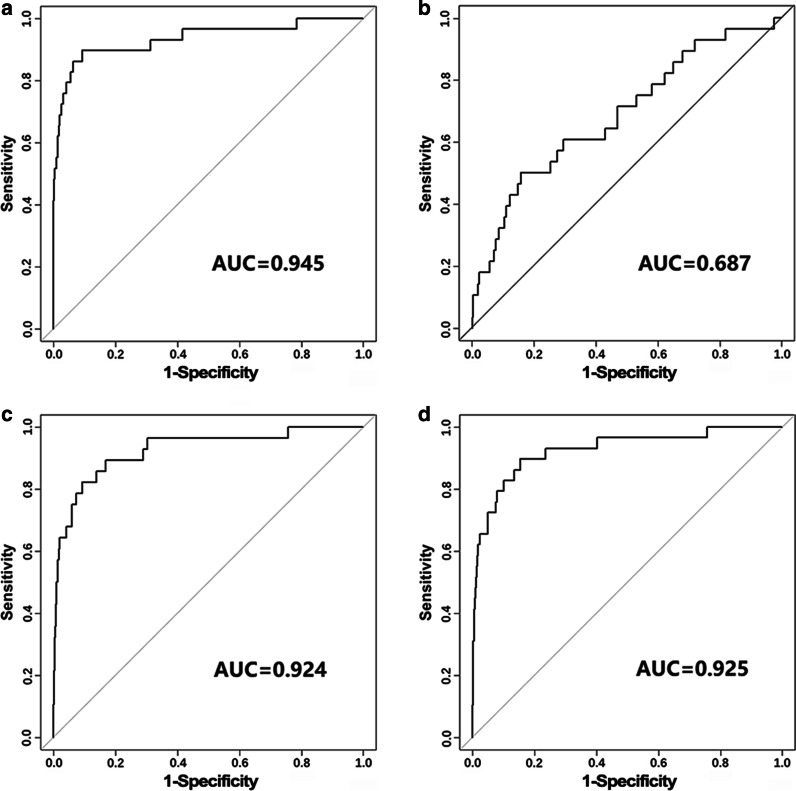
ROC curves of **a** PEP, **b** ToppNet, **c** random walk, and **d** k-step Markov

The performance of the PEP method was compared with that of other state-of-the-art network-based prioritization methods. The comparison was first conducted between the PEP method and ToppNet of the ToppGene Suite, Random Walk and k-step Markov of LynxKB, which exploited the topological information (Fig. 3). Results of LOOCV showed that the PEP method had the highest AUC value, while the AUC values for the k-step Markov and Random Walk applying random walk were a little inferior, and that for ToppNet were the lowest.

**Fig. 4:**
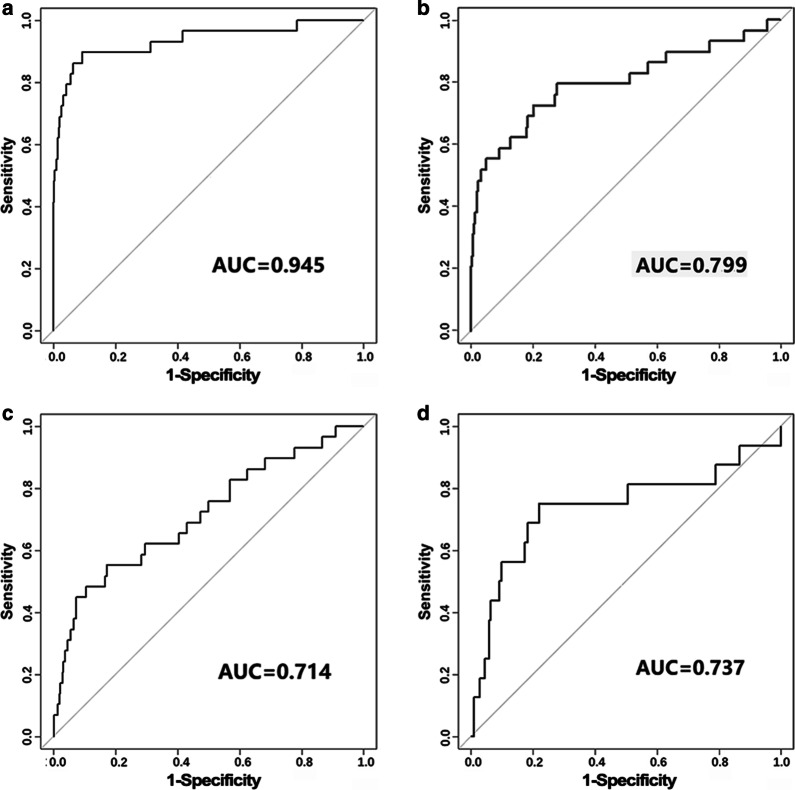
ROC curves of **a** PEP, **b** our previous work, **c** ToppGene, and **d** Fun_L

The PEP method was then compared to our previous work in [12], ToppGene and FUN-L exploiting functional information (Fig. 4). The AUC values for the latter three methods were between 0.7 and 0.8, which were all less than that for the PEP method.

These results indicated that the performance of the PEP method was better than that of other existing methods using either topological information or expression information.

### COPD Candidate gene prioritization

Genes in the COPD PPI network were prioritized based on their disease risk scores in descending order. The top-ranked candidate genes were supposed to be more relevant to COPD. To prove their relationships, a literature review and functional enrichment analysis were conducted for the top 200 candidate genes (Additional file 1).

#### Literature validation

After searching in the NCBI PubMed database (https://www.ncbi.nlm.nih.gov/pubmed), nearly half of the top 200 candidate genes were validated as being associated with COPD. It is worth highlighting that the validation rate for higher-ranked genes was higher than that for lower-ranked genes. That is, 50% of the top 50, 44% of the top 100, 40% of the top 150, and 37% of the top 200 candidate genes were validated.

For the first ranked candidate gene, UGT1A1, its low expression was found to play a protective role in COPD since several studies has found that the enzyme uridine diphosphate glucuronosyltransferase polypeptide 1A1, encoded by gene UGT1A1, was responsible for clearing bilirubin from the blood, whereas higher bilirubin concentrations were associated with a lower risk of acute exacerbations of COPD [21–23]. The serum levels of BDNF (Rank: 2) (but not concentrations of platelets in the peripheral blood) were significantly elevated at all stages of COPD as compared to controls [24]. SHC1 (Rank: 3) was significantly decreased in alveolar epithelial cells in COPD patients. Thus, it could reduce the risk of lung diseases [25, 26]. The up-regulation of CREB1 (Rank: 5) activated pro-inflammatory HSP60 in bronchial epithelial cells, as observed in severe COPD patients compared to control smokers and non-smokers [27]. Airway levels of MUC2 (Rank: 9) were decreased in patients with severe COPD colonized by potentially pathogenic micro-organisms [28]. These genes participated in the disease regulation process and could be main factors in the pathogenesis of COPD.

#### Functional enrichment analysis

**Fig. 5:**
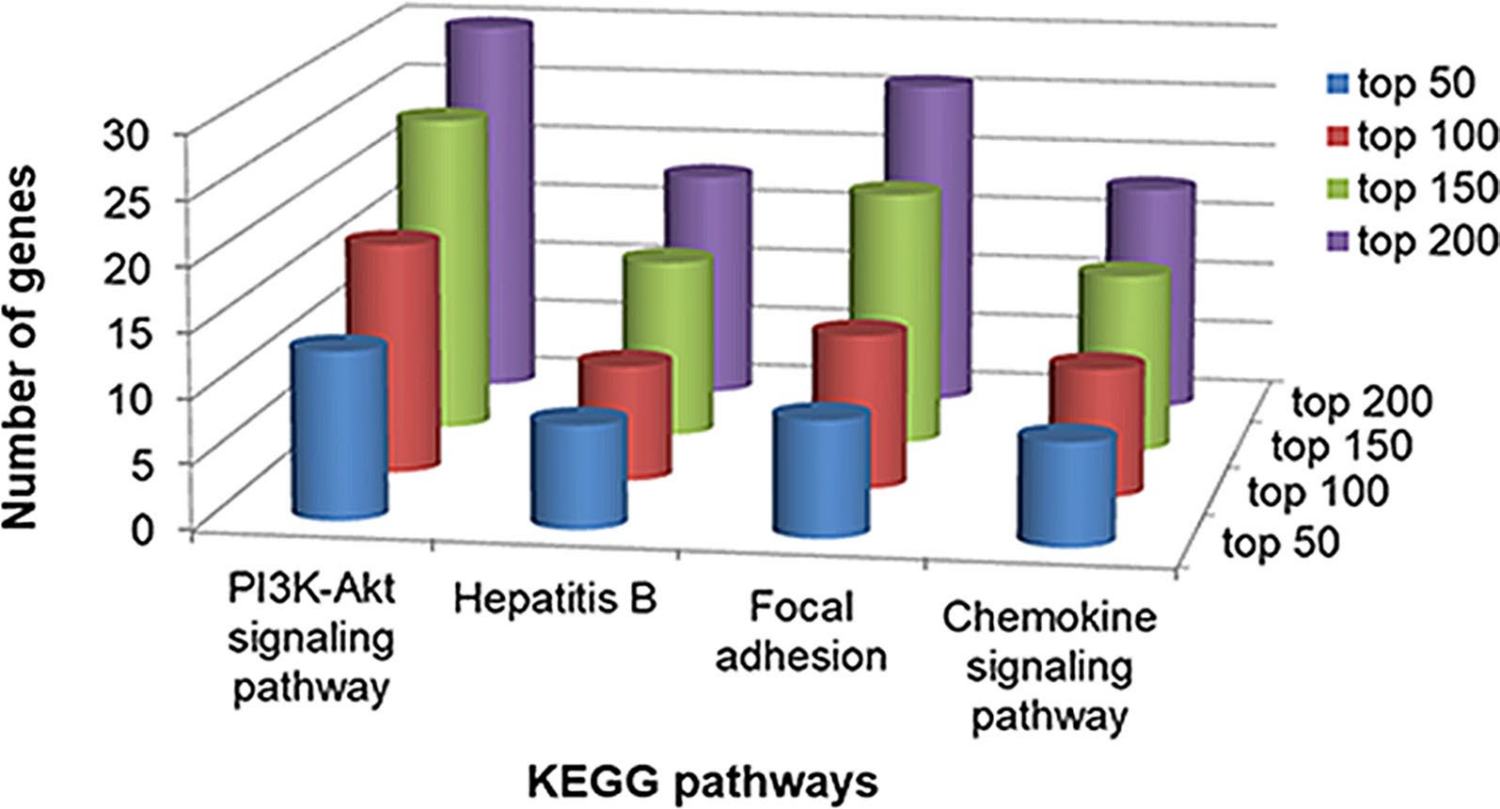
KEGG pathways significantly enriched by the top 50, 100, 150 and 200 candidate genes

Functional enrichment analysis was performed for the 200 candidate genes using DAVID. The top 200 candidate genes were significantly enriched in 37 COPD-related KEGG pathways, especially four pathways that were significantly enriched in by the top 50 candidate genes (Fig. 5). The “PI3K-Akt signaling pathway” was required for Sirtuin 1 induction by endoplasmic reticulum stress and exacerbated the COPD [29]. The increases in “Focal adhesion” expression affected the proliferation of apical cells behind the wound edge related to some respiratory diseases, such as COPD [30]. The loss of epithelial anion transport in COPD is correlated with the increased inflammation driven by the release of chemokines regulated by the “Chemokine signaling pathway” and subsequent immune cell infiltration of the respective organs [31]. Few studies showed the association of the “Hepatitis B pathway” with COPD. However, genes in the pathway were also found to be involved in the COPD-related “PI3K-Akt signaling pathway” and lung-related pathways, such as “Influenza A” and “Tuberculosis”. Additionally, a study of the efficiency of the anti-hepatitis B vaccination in adults with COPD suggested that the pathway could influence the COPD process under treatment with Affonoleikin [32]. These results implied the relationship between the “Hepatitis B pathway” and COPD to some extent, while further studies are still needed.

**Fig. 6:**
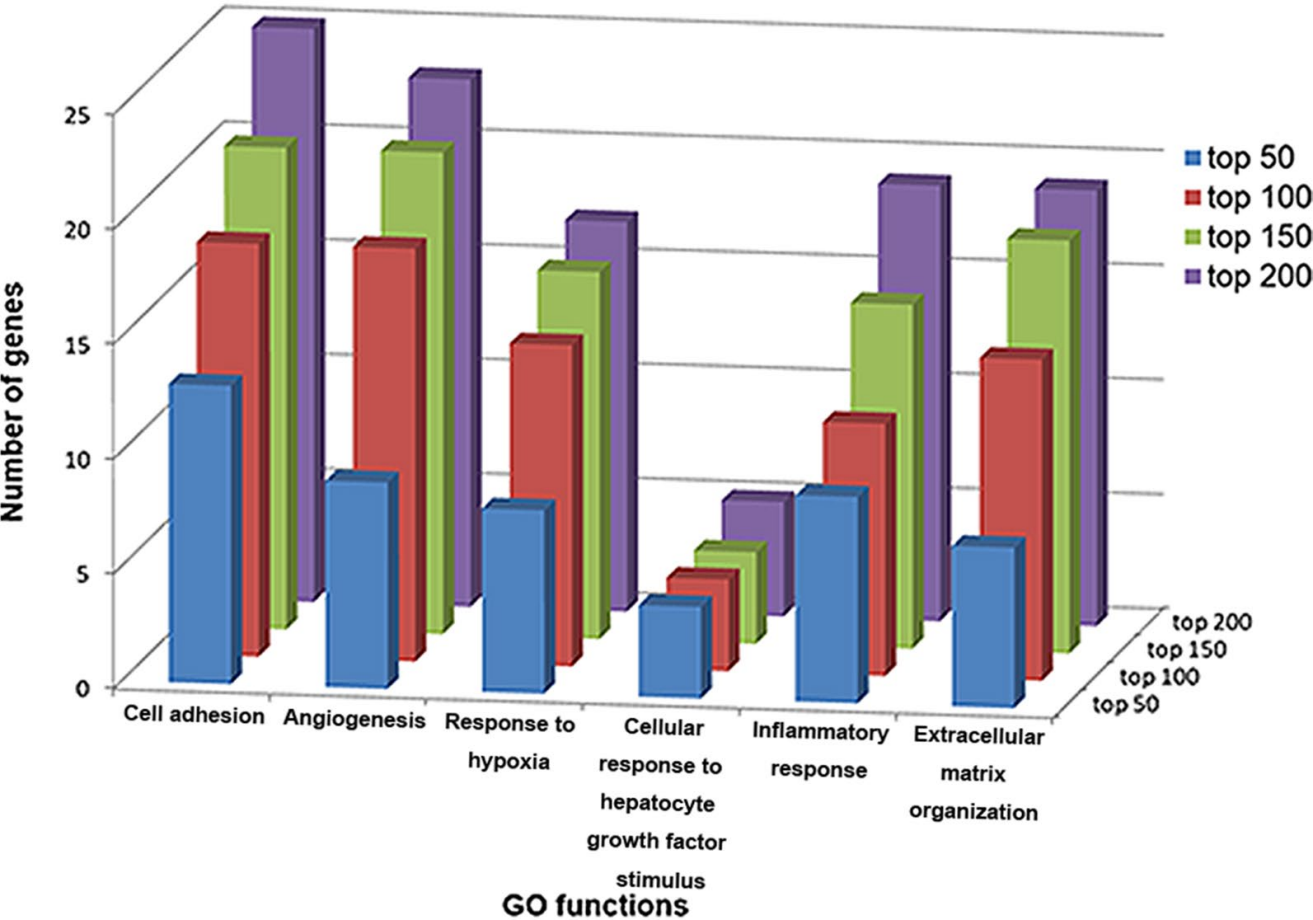
GO functions significantly enriched by the top 50, 100, 150 and 200 candidate genes

Moreover, 33 COPD-related GO functions were significantly enriched in by the top 200 candidate genes (Fig. 6). For example, impairing “cell adhesion” could alter the function of airway epithelial cells. These changes contributed to local inflammation, which led to lung function decline and increased susceptibility to COPD [33]. “Angiogenesis” was observed to be significantly decreased among COPD patients versus controls. This suggested the possibility of blunted “angiogenesis” in COPD patients, who showed impaired training-induced blood pressure adaptation related to a change in muscle capillarizatio [34, 35]. Hepatocyte growth factor was involved in the pathogenesis of various lung diseases as it was significantly higher in COPD patients compared to control patients. Hence, the “cellular response to hepatocyte growth factor stimulus” might be relevant for tissue repair in COPD [36]. The remodeling of the “extracellular matrix organization” is a common feature in lung diseases such as COPD [37].

These results showed that the top 200 candidate genes prioritized by the PEP method could be enriched in COPD-related pathways and functions. It was also indicated that these genes might be potential disease genes of COPD.

### Independent data validation

**Fig. 7:**
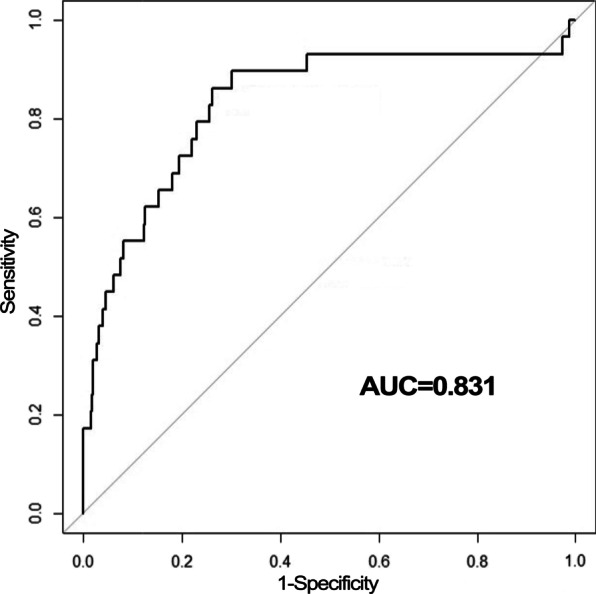
The ROC curve of the PEP method for GSE76925

To test the applicability of our method for other microarray data, the PEP method was applied to another independent microarray data GSE76925, which contained 40 normal samples and 111 COPD patients. The AUC value of LOOCV was 0.831, which demonstrated the good performance of the PEP method (Fig. 7).

As for GSE57148, four sets of samples were randomly selected with an equal number of disease samples and normal samples from GSE76925. The randomization process was repeated 100 times. The AUC values of LOOCV were also used to evaluate their performance. The robustness of the PEP method was also confirmed, since all AUC values were larger than 0.83 for different sample sizes.

All the above results suggested that the PEP method was effective and robust for both RNA-seq and microarray data.

## Discussion

The expression information of normal and disease samples can reveal dynamic changes between these statuses. In this paper, disease candidate genes were prioritized based on the disease risk scores from a COPD PPI network using expression information by a newly proposed method PEP. The PEP method using expression information in the COPD PPI network had good robustness, since the AUC values using LOOCV were all larger than 0.86 for different sample sizes randomly selected from the original profile. The method was also superior to other existing methods exploiting either topological or functional information.

Different values for parameters $h$ and $k$ had certain influence on the results of the PEP method. After investigating the performance of different values, $h$ = 1 and $k$ = 0.7 were found to be the optimal parameters, demonstrating that the COPD disease genes and candidate genes were of equal importance in the COPD PPI network, and the vertex and edge information should be considered simultaneously.

The top 200 candidate genes prioritized with optimal parameters were confirmed to be correlated with COPD through a literature review and functional enrichment analysis. As a result, the top 200 candidate genes might be potential disease genes in the diagnosis and treatment of COPD. In order to test the potential ability of the top-ranked candidate genes acting as markers, they were used as classification features to classify samples of the original profile by applying the support vector machine (SVM) method with a linear kernel. The performance was assessed by comparing the AUC values for the COPD disease genes, the top 29 (the same number as the COPD disease genes) candidate genes, and four groups of 50 genes from the top 200 candidate genes (the top 50, Rank 51–100, 101–150 and 151–200). It was demonstrated that different classification features were all effective to classify samples (Table 3). The AUC value of the top 29 candidate genes was higher than the COPD disease genes, indicating a stronger discriminative power. The AUC value for the top 50 candidate genes had the best classification performance. Therefore, top-ranked genes prioritized by the PEP method could be used as markers to identify disease samples.

**Table 3:** AUC values of the classification performance of different classification features

Classification feature	AUC value	Classification feature	AUC value
COPD disease genes	0.837	Rank 51–100 genes	0.881
top 29 genes	0.846	Rank 101–150 genes	0.810
top 50 genes	0.882	Rank 151–200 genes	0.745

Our study has some limitations. The computation process of the PEP method was complicated, which made it inappropriate to be performed as a web tool at present. The code need to be further optimized so that a web tool could be constructed to facilitate the use of other researchers. Additionally, top 200 candidate genes were verified using literature reviews, which might not be objective enough. Further downstream validation experiments and functional studies is needed to reveal their biological relevance with COPD.

## Conclusions

To sum up, the PEP method was effective and robust in prioritizing disease candidate genes using expression information from sequencing or microarray data. Therefore, the top-ranked candidate genes of the PEP method or their significantly-enriched functions were verified to be related to COPD. These genes could also classify COPD and normal samples effectively. In addition, the PEP method could provide new insights to the research of prioritizing disease candidate genes and identifying potential makers of diseases, and could be applied to other complex diseases.

## Supplementary Information

**Additional file 1**. The list of top 200 candidate genes.

## Data Availability

The dataset GSE57148 analyzed during the current study are available in the Gene Expression Omnibus (GEO) database, https://www.ncbi.nlm.nih.gov/geo/query/acc.cgi?acc=GSE57148. Other data that support the findings of this study are available from the corresponding author upon reasonable request.
