# Supplementary material for: Candidate gene prioritization for chronic obstructive pulmonary disease using expression information in protein–protein interaction networks
Source: BMC Pulm Med. 2021 Sep 4;21:280. doi: 10.1186/s12890-021-01646-9 (PMC8418003; doi:10.1186/s12890-021-01646-9)
Supplement: Supplementary file 1 — Additional file 1. The list of top 200 candidate genes. [file 12890_2021_1646_MOESM1_ESM.docx]

**Table S1. The list of top 200 candidate genes.**

| Rank | Gene | Disease risk score |
| --- | --- | --- |
|  | UGT1A1 | 0.00450 |
|  | BDNF | 0.00186 |
|  | SHC1 | 0.00182 |
|  | CSRP3 | 0.00177 |
|  | CREB1 | 0.00176 |
|  | KRT16 | 0.00174 |
|  | COL18A1 | 0.00174 |
|  | UGT1A7 | 0.00173 |
|  | MUC2 | 0.00152 |
|  | THBS2 | 0.00147 |
|  | RHOA | 0.00146 |
|  | MYC | 0.00145 |
|  | SPP1 | 0.00143 |
|  | GTF2H3 | 0.00141 |
|  | CAV1 | 0.00136 |
|  | SERPINE2 | 0.00135 |
|  | EDN1 | 0.00135 |
|  | MLN | 0.00133 |
|  | ERCC2 | 0.00132 |
|  | DDX41 | 0.00126 |
|  | IL10 | 0.00125 |
|  | IGFBP3 | 0.00124 |
|  | MYBPC1 | 0.00124 |
|  | TEK | 0.00122 |
|  | RELA | 0.00120 |
|  | HDAC9 | 0.00118 |
|  | COL5A1 | 0.00116 |
|  | IL3 | 0.00116 |
|  | HSPG2 | 0.00115 |
|  | MAPK9 | 0.00113 |
|  | TUSC5 | 0.00112 |
|  | TLR4 | 0.00112 |
|  | JAK2 | 0.00111 |
|  | CXCL12 | 0.00111 |
|  | LEP | 0.00108 |
|  | GPNMB | 0.00108 |
|  | PCNA | 0.00106 |
|  | CTRB1 | 0.00105 |
|  | WWOX | 0.00103 |
|  | HIF1A | 0.00103 |
|  | CDKN2A | 0.00102 |
|  | ADAM12 | 0.00102 |
|  | COL6A1 | 0.00102 |
|  | CXCR3 | 0.00102 |
|  | BCAR1 | 0.00101 |
|  | FGFR1 | 0.00099 |
|  | ENG | 0.00099 |
|  | IKBKB | 0.00099 |
|  | NOTCH4 | 0.00099 |
|  | ACAN | 0.00098 |
|  | ANGPT1 | 0.00098 |
|  | FOXP3 | 0.00097 |
|  | COL11A1 | 0.00096 |
|  | KDM2A | 0.00095 |
|  | EPHB2 | 0.00095 |
|  | SH3PXD2B | 0.00095 |
|  | HSPB2 | 0.00095 |
|  | NOX5 | 0.00093 |
|  | FOSL1 | 0.00092 |
|  | CCKAR | 0.00092 |
|  | TIE1 | 0.00092 |
|  | NINJ1 | 0.00092 |
|  | ADAM9 | 0.00090 |
|  | TGM6 | 0.00089 |
|  | NOS1 | 0.00087 |
|  | ADIPOQ | 0.00087 |
|  | PROM1 | 0.00087 |
|  | SELE | 0.00087 |
|  | ROCK1 | 0.00086 |
|  | ACVRL1 | 0.00086 |
|  | BLOC1S1 | 0.00085 |
|  | FGFR2 | 0.00084 |
|  | ANGPT2 | 0.00084 |
|  | COL4A1 | 0.00084 |
|  | FBN1 | 0.00084 |
|  | SERPINB5 | 0.00083 |
|  | RECK | 0.00083 |
|  | SI | 0.00083 |
|  | TGFBI | 0.00083 |
|  | LGALS7B | 0.00082 |
|  | UBC | 0.00082 |
|  | AGT | 0.00082 |
|  | SPSB1 | 0.00082 |
|  | UGT2B17 | 0.00082 |
|  | CD44 | 0.00081 |
|  | SAP25 | 0.00081 |
|  | MATN2 | 0.00080 |
|  | TGM7 | 0.00080 |
|  | OR1L1 | 0.00080 |
|  | KLF8 | 0.00080 |
|  | PTGS2 | 0.00079 |
|  | FOXO1 | 0.00078 |
|  | SOD2 | 0.00078 |
|  | ECE1 | 0.00078 |
|  | SMAD4 | 0.00078 |
|  | TBX10 | 0.00078 |
|  | AR | 0.00078 |
|  | HIST1H4A | 0.00077 |
|  | OGT | 0.00076 |
|  | ADCY5 | 0.00076 |
|  | SH3PXD2A | 0.00076 |
|  | AKT2 | 0.00076 |
|  | ANKRD50 | 0.00075 |
|  | TLE3 | 0.00075 |
|  | ACTN1 | 0.00075 |
|  | TGFBR1 | 0.00075 |
|  | PRDM10 | 0.00075 |
|  | SLC2A4 | 0.00075 |
|  | CHD9 | 0.00075 |
|  | COL4A2 | 0.00075 |
|  | MAPK11 | 0.00075 |
|  | BDKRB2 | 0.00074 |
|  | HSF1 | 0.00074 |
|  | NRAS | 0.00073 |
|  | OCLN | 0.00073 |
|  | ZNF461 | 0.00073 |
|  | ARRB1 | 0.00072 |
|  | LAMA3 | 0.00072 |
|  | GATA2 | 0.00071 |
|  | UGT2B11 | 0.00071 |
|  | HSD3B1 | 0.00071 |
|  | BMP2 | 0.00071 |
|  | UGT1A4 | 0.00071 |
|  | RELN | 0.00070 |
|  | HIF3A | 0.00070 |
|  | CYP2C19 | 0.00070 |
|  | MAPK8 | 0.00069 |
|  | OBSCN | 0.00069 |
|  | MAPK7 | 0.00069 |
|  | JUNB | 0.00069 |
|  | SUDS3 | 0.00069 |
|  | ELAVL1 | 0.00068 |
|  | TEAD4 | 0.00068 |
|  | MDM2 | 0.00067 |
|  | HTR3D | 0.00067 |
|  | SAP30BP | 0.00067 |
|  | NFKB2 | 0.00067 |
|  | CAMK2D | 0.00067 |
|  | FAU | 0.00066 |
|  | CCL7 | 0.00066 |
|  | JUN | 0.00066 |
|  | ILF3 | 0.00066 |
|  | KDM5A | 0.00066 |
|  | ITGAM | 0.00066 |
|  | ARHGAP23 | 0.00065 |
|  | BCL2L1 | 0.00065 |
|  | ACTB | 0.00065 |
|  | EDNRB | 0.00065 |
|  | HLA-E | 0.00065 |
|  | LTBP1 | 0.00065 |
|  | TF | 0.00064 |
|  | GJA4 | 0.00064 |
|  | SMAD5 | 0.00063 |
|  | DPYD | 0.00063 |
|  | TP53INP2 | 0.00063 |
|  | HIST1H2BM | 0.00063 |
|  | RPS6KA1 | 0.00062 |
|  | SPHK1 | 0.00062 |
|  | FAT1 | 0.00062 |
|  | CDH5 | 0.00062 |
|  | PIK3CG | 0.00062 |
|  | SOS1 | 0.00061 |
|  | RPS6KA2 | 0.00061 |
|  | ADAMTS4 | 0.00061 |
|  | SLC7A1 | 0.00061 |
|  | PDGFC | 0.00061 |
|  | GSTA4 | 0.00061 |
|  | TGFB2 | 0.00061 |
|  | CXCR2 | 0.00061 |
|  | SMAD9 | 0.00061 |
|  | DICER1 | 0.00060 |
|  | OPN4 | 0.00060 |
|  | GSTP1 | 0.00060 |
|  | LDLR | 0.00060 |
|  | KLF4 | 0.00060 |
|  | FYN | 0.00059 |
|  | HNF4A | 0.00059 |
|  | ATP2A2 | 0.00059 |
|  | MUC5B | 0.00059 |
|  | PROS1 | 0.00059 |
|  | HIST1H2BB | 0.00059 |
|  | PAX2 | 0.00059 |
|  | SIPA1 | 0.00059 |
|  | APOC3 | 0.00059 |
|  | CAMK2A | 0.00058 |
|  | LRPAP1 | 0.00058 |
|  | FGG | 0.00058 |
|  | NPHS1 | 0.00058 |
|  | REL | 0.00058 |
|  | BCL2 | 0.00057 |
|  | CLDN5 | 0.00057 |
|  | ABCB11 | 0.00057 |
|  | FER1L6 | 0.00057 |
|  | BAZ2A | 0.00057 |
|  | GSK3B | 0.00057 |
|  | AKAP7 | 0.00057 |
|  | ACACB | 0.00057 |
|  | EGR3 | 0.00057 |
|  | PIM2 | 0.00057 |
|  | PCSK7 | 0.00056 |
